# Anisotropy in the Liquefaction Resistance of Fibre Reinforced Sand

**DOI:** 10.3390/ma16216959

**Published:** 2023-10-30

**Authors:** Xidong Zhang, Yan Zhuang, Zhen’ang Wang, Changxing Yang, Shunlei Hu

**Affiliations:** 1 Shanxi Key Laboratory of Civil Engineering Disaster Prevention and Control, Institute of Geotechnical & Underground Engineering, School of Civil Engineering, Taiyuan University of Technology, Taiyuan 030024, China; zhangxidong@tyut.edu.cn (X.Z.); wangzhenang116@163.com (Z.W.); cxyang_o@163.com (C.Y.); 2 Key Laboratory of Concrete and Prestressed Concrete Structures of Ministry of Education, School of Civil Engineering, Southeast University, Nanjing 210096, China; hushunlei97@163.com

**Keywords:** fibre reinforcement, sand, liquefaction resistance, anisotropy, effective stress path

## Abstract

Adding discrete fibres to sand has been seen as a feasible technique to improve sand’s strength as well as liquefaction resistance. Considering the anisotropic distribution of fibre orientations, the anisotropy in the liquefaction resistance of the reinforced sand is also introduced using fibres. Here, the triaxial compression and extension test results of unreinforced and fibre-reinforced sand in different density states are provided, from which the anisotropy in the liquefaction resistance of fibre-reinforced sand is demonstrated. Fibre reinforcement improves the liquefaction resistance of sand by introducing both the densifying effect and the confining effect. The inclusion of fibres increases both the slope and the intercept of the strength envelope in comparison with the unreinforced sand under triaxial compression, while the strength envelope is not affected by fibres under triaxial extension. Stress contribution of fibres makes the ESP of the composite under undrained loading reverse its direction to develop even though the phase transformation is absent. The stress ratio initiating the ESP reversal is irrespective of the fibre content but dependent on the density state under triaxial compression. Under triaxial extension, the stress ratio initiating the ESP reversal remains the same in the samples with varied density states and fibre contents. The mechanism correlating to the strength envelope and ESP reversal of the fibre-reinforced sand was demonstrated following a rule of mixture based constitutive modelling framework. By introducing an alternatively defined pore pressure ratio that incorporates the stress contribution of fibres, the liquefaction state of the fibre reinforced sand is reasonably assessed. Liquefaction remains absent in the sand once the fibres are mixed. The anisotropy in the liquefaction resistance of fibre-reinforced sand arises, as the predominant role played by the fibres to suppress the liquefaction is different when varied loading paths are involved, which is sourced from the anisotropic distribution of fibre orientations.

## 1. Introduction

Mixing discrete flexible fibres into sands is a promising soil-reinforcing technique that has been widely focused on by geotechnical engineers in recent decades. Fibre-reinforcing technology originates from the principles of vegetation root systems strengthening near-surface soils [1,2]. The benefits of fibre reinforcement may include strengthening soils, especially granular soils, making the soil more failure resistant, and in some cases improving the liquefaction resistance of sands [1,3,4]. Direct shear tests were broadly adopted in early experimental investigations on fibre-reinforced sand. It was generally concluded that the presence of fibres increased the peak shear strength of sand and also limited the post-peak reduction of the shear resistance [5,6,7,8]. Muir Wood [9] argued that under direct shear conditions, a tensioned fibre not only induces additional shear stress but also imposes additional normal stress on the sample, thus enhancing the shear resistance of sand. The beneficial effects of fibre reinforcement on the strength of sand have been further demonstrated in triaxial tests. There are too many academic studies to make a complete review here. Consistent results have been reported in different studies, indicating that the presence of fibres increases the shear strength of sand. However, to what extent the shear strength of sand is improved by the addition of fibres may depend on the fibre properties (i.e., fibre length, fibre diameter, fibre content, elastic modulus of fibres, fibre orientation), soil properties (i.e., particle size, particle shape, gradation, density state), as well as the confining stress acting on the fibre–sand composite [10,11,12,13,14,15,16,17,18,19,20,21,22].

The studies on liquefaction responses of fibre-reinforced sand seem to be less frequent than those on the drained strength characteristics. Undrained cyclic triaxial tests, in some cases, cyclic simple shear tests and cyclic hollow torsion shear tests, have been adopted to examine the effect of fibre reinforcement on the liquefaction resistance of sand. It has been found that mixing fibres into sand suppresses the build-up of excess pore pressure and also decelerates the accumulation of cyclic strain, which convinces researchers that the liquefaction resistance of sand can be improved by fibres [1,23,24,25,26,27,28,29]. Fibre reinforcement also shows a good ability to suppress the static liquefaction of sand. Once fibres are added to loose sand, a strain-softening response is prevented from developing, and the static liquefaction remains absent [30,31]. Shaking table tests provide a means to improve the understanding of earthquake loading-induced liquefaction [32,33]. Maheshwari et al. [34] investigated the liquefaction resistance of Solani sand reinforced using different types of fibres through shaking table tests. The test results showed that adding fibres increased liquefaction resistance because fibre reinforcement decreased the maximum excess pore pressure (EPP) built up during the shaking. However, it was found that the effect of fibre reinforcement on suppressing the liquefaction potential of sand was limited during shaking table tests once the laminated shear stack was adopted [3,35].

Fibres are mostly the unidirectional and tension-resistant element. They can only carry tension loads along their directions while their bending stiffness can be ignored. Therefore, fibres may lead to strength anisotropy in the sand once the fibre orientations are not randomly distributed. Gray and Ohashi [5] reported the dependence of the sand strength on the fibre orientation through the early conducted direct shear tests. It was observed that the greatest strength improvement occurred when the fibres were orientated with an angle of 60° with respect to the shear surface (i.e., horizontal). Michalowski and Cermak [36] found that the strength of the fibre reinforced sand is largely affected by the fibre orientations. Under triaxial compression, the horizontally orientated fibres yielded the greatest strength in the reinforced samples, while the randomly orientated fibres provided a lesser strength increment. The vertically orientated fibres, however, imposed an adverse effect on the composite’s strength.

It is not difficult to see that the strength anisotropy of fibre-reinforced sand is predominantly controlled by the nonuniformity of the fibre orientation distribution [17]. With the well-established fabrication method for fibre-reinforced samples in experimental studies, i.e., moist tamping and moist vibration, a significant anisotropic fibre orientation distribution is produced. Fibres tend to orientate sub-horizontally being axisymmetric with respect to the axis which is normal to the bedding plane which is usually horizontal for the vertically tamped samples [37,38]. The anisotropic distribution of fibre orientations induces the mechanical response of the reinforced sand being loading path-dependent. The reinforced sand behaves differently under drained compression and extension. The strength of the sand is significantly increased by the fibres under drained triaxial compression while the strength of the sand is almost indifferent to the addition of fibres [20,39]. The strength anisotropy of fibre-reinforced sand was recently illustrated by performing the hollow cylinder torsional shear (HCTS) tests. The strength of fibre-reinforced sand is largely affected by the direction of the major principal stress [40,41,42]. Roughly, a rise in inclination angle of the major principal stress with respect to the deposition direction, which is usually horizontal, causes a reduction in strength.

Regarding fibre reinforcement adopted as a technique to mitigate the liquefaction potential of sand, the anisotropy of liquefaction resistance possibly induced by the nonuniformity orientations of fibres has been seldom investigated. Most studies focusing on the liquefaction responses of fibre-reinforced sand experimentally show the beneficial effect of fibres on increasing the liquefaction resistance of sand. In-depth analysis of the mechanism that fibres suppress liquefaction potential is usually absent. Zhang and Russell [31] demonstrated how applied loads were distributed and shared by fibres, sand skeleton and pore water under undrained loading. How tensioned fibres alter the effective stress paths (ESPs) experienced by the sand skeleton and thus alter the liquefaction resistance of sand was also illustrated, in which the anisotropy of liquefaction resistance of fibre reinforced sand was, however, not involved. In this study, the anisotropic liquefaction resistance of fibre-reinforced sand is demonstrated by performing triaxial tests on it under compression and extension conditions. Two effects, i.e., a confining and densifying effect, introduced by the fibres, that may potentially increase the liquefaction resistance of sand were investigated. In particular, how confining and densifying effects evolve under different loading paths and how these two effects induce the anisotropy of liquefaction resistance in fibre-reinforced sand are discussed.

## 2. Test Materials and Test Program

### 2.1. Test Materials and Sample Preparation

**Figure 1:**
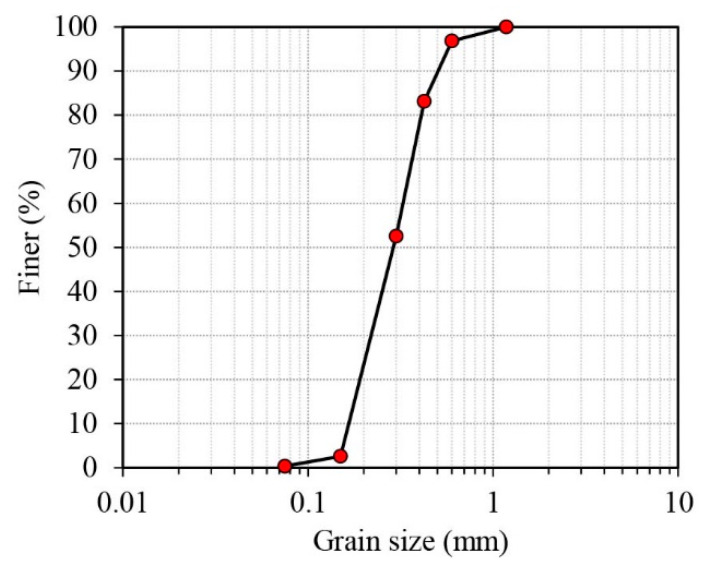
The particle size distribution of Sydney sand.

In this study, Sydney sand was used in all tests. The particle size distribution determined using sieve analysis is shown in Figure 1. The index properties of the Sydney sand are provided in Table 1. The sand is poorly graded, with subrounded to rounded particle shapes.

**Figure 2:**
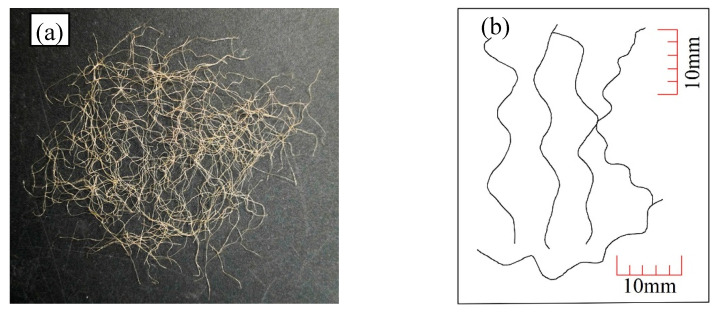
Loksand fibres used in the tests: (**a**) the general view and (**b**) a sketch of 5 single fibres.

In the reinforced samples, Loksand fibres manufactured by Drake Extrusion, UK, were used to provide reinforcement. The general view of the fibres is shown in Figure 2. Every single fibre has a length of 35 mm and a diameter of 0.1 mm, with an aspect ratio (length/diameter) of 350. The fibres are fine but can be easily separated into monofilaments. Every single fibre has a tensile strength of 225 MPa and an elastic modulus of 900 MPa. The specific gravity of the fibres is 0.91.

All samples in this study were prepared using the moist tamping method, which involved two main steps, i.e., fibre–sand mixing and compaction. The sand was first wetted to a water content of 10% by mixing the de-aired water into dry sand. Increasing the water content of the sand significantly avoided the flotation of fibres during the mixing, facilitating the production of high-quality fibre–sand mixtures. The fibres were mixed into the wet sand batch by batch, and in each mixing, a small portion of the target number of fibres was added. Before mixing the fibres into the sand, the fibres were manually separated into monofilaments. The procedures described above largely ensure the uniform distribution of fibres in the sand skeleton and a homogenous fibre–sand composite was made. The wet sand (for unreinforced samples) or fibre–sand mixtures (for reinforced samples) were then gently transferred into the mould and compacted to fabricate the sample. All samples were cylindrical with both diameter and height of 50 mm. The samples were tamped in four equal layers and each layer had a thickness of 12.5 mm. In this study, fibres were introduced when keeping the mass of the dry sand constant. The amount of fibre added to the samples was quantified using the fibre content (FC), which was defined to be the ratio of the mass of fibres (*M_f_*) over the dry mass of sand (*M_s_*), i.e., FC = *M_f_*/*M_s_* × 100%. The fibre contents adopted in this study included 0, 0.25% and 0.5%. The samples were placed in a freezer together with the mould for at least 12 h before they were taken out from the mould to be tested.

### 2.2. Test Program

**Figure 3:**
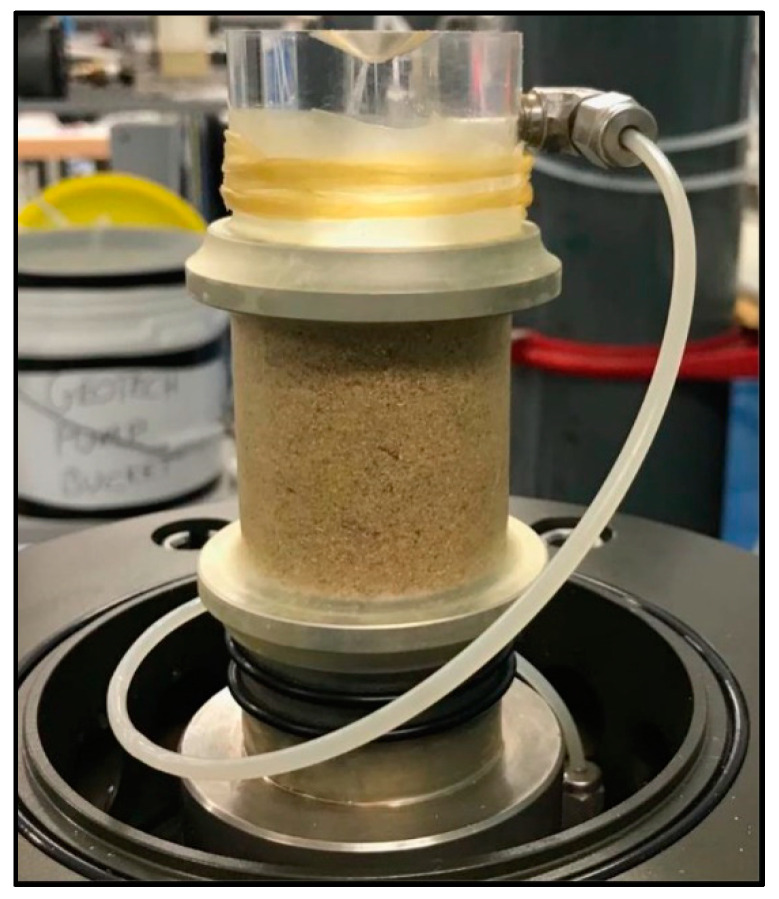
The sample positioned in the triaxial cell with enlarged and lubricated end platens.

In this study, load-controlled triaxial tests, including triaxial compression and extension, were performed on both unreinforced and fibre-reinforced sand samples in varied density states, including very loose (VL) state, loose (L) state and medium dense (MD) state. To maintain a uniform shape of the sample during loading, enlarged and lubricated end platens were used in all tests (Figure 3). The enlarged and lubricated ends enable the use of samples with a height to diameter ratio less than 2 in the triaxial tests [43,44]. Samples with a height-to-diameter ratio of 1 together with enlarged and lubricated ends have been widely adopted by others [30,45,46] to investigate the liquefaction responses of sand and reinforced sand in triaxial tests, from which reliable results were obtained. The samples were saturated using the CO_2_ method together with a back pressure of up to 350 kPa. The samples were deemed to be saturated once the B-value (Skempton’s coefficient) attained at least 0.99. The samples were isotropically consolidated under confining stresses of 50 kPa, 100 kPa and 200 kPa. The tests performed in this study are listed in Table 2. The void ratios of samples after consolidation were calculated when fibres were treated as being part of the solid. *p_c_* in Table 2 represents the net confining pressure in excess of the back pressure.

## 3. Test Results

To maintain brevity, only the results of the triaxial compression and extension tests on both unreinforced and fibre reinforced samples in different density states under *p_c_* = 100 kPa are illustrated. For the test results, triaxial notations were adopted. The total mean stress (in excess of back pressure) and the total deviator stress acting on the fibre–sand composite have been denoted, respectively, as p and q, which are defined by Equation (1), where $\sigma_{a}$ and $\sigma_{r}$, respectively, represent the axial and radial stress. The volumetric strain (i.e., $\varepsilon_{v}$) and the shear strain (i.e., $\varepsilon_{q}$) of the sample are given by Equation (2), in which $\varepsilon_{a}$ and $\varepsilon_{r}$ are the axial and radial strains. In the undrained loading tests, $\varepsilon_{v}$ remains 0 while $\varepsilon_{q}$ = $\varepsilon_{a}$.

(1) $$p=\frac{\sigma_{a}+{2\sigma }_{r}}{3};\;\;\;\;\;\;q=\sigma_{a}-\sigma_{r}$$

(2) $$\varepsilon_{v}=\varepsilon_{a}+2\varepsilon_{r};\;\;\;\;\;\;\varepsilon_{q}=\frac{2}{3}\left(\varepsilon_{a}{-\varepsilon }_{r}\right)$$

To distinguish the effective stress imposed on the sand skeleton from that on the composite, the effective mean stress and deviator stress of the composite are, respectively, denoted by $p^{*}$ and $q^{*}$, while the effective mean stress and deviator stress of the sand skeleton are, respectively, indicated by $p^{\prime }$ and $q^{\prime }$. In the undrained loading condition, the excess pore pressure (EPP) is denoted by u, $p^{*}=p-u$ and $q^{*}=q$. The mean stress and deviator stress carried by the fibres are, respectively, denoted as $p_{f}$ and $q_{f}$. The conventional EPP ratio, denoted as $r_{u}$, is adopted to present the build-up in EPP, being defined as $r_{u}$ = u/$p_{c}$.

### 3.1. Drained Tests

**Figure 4:**
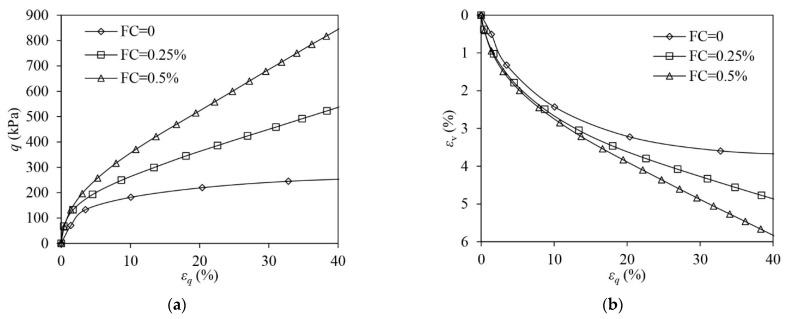
The test results of very loose samples under drained triaxial compression: (**a**) deviator stress–shear strain; (**b**) volumetric strain–shear strain.

Figure 4 shows the test results of both unreinforced (FC = 0) and fibre-reinforced (FC = 0.25%, FC = 0.5%) sand samples in VL states under drained triaxial compression. Strain-hardening responses are experienced by all samples. When compared with the unreinforced sand, the fibre-reinforced sand attains a greater strength at a given shear strain. Adding more fibres further increases the strength. When $\varepsilon_{q}$ = 40%, the strength of the reinforced sample with FC = 0.5% increases by 232.2% more than the unreinforced sample. Under triaxial compression, both unreinforced and fibre-reinforced samples show suppressed volumetric contraction due to their VL states. The volume of the sample contracts more when fibres are added.

**Figure 5:**
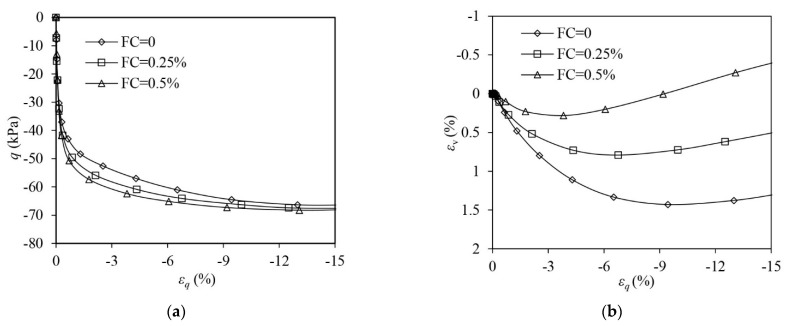
The test results of very loose samples under drained triaxial extension: (**a**) deviator stress–shear strain; (**b**) volumetric strain–shear strain.

Figure 5 illustrates the test results on the samples in VL states under drained triaxial extension. Strain-hardening responses are also exhibited in all samples. Fibres seem to be incapable of improving the stress–strain response of sand. Adding fibres into sand only slightly increases the strength. The effect of fibres on volumetric behaviour under triaxial extension is opposite to when under triaxial compression. Reinforced samples show more volumetric dilation than the unreinforced ones.

### 3.2. Undrained Tests

#### 3.2.1. VL Samples

**Figure 6:**
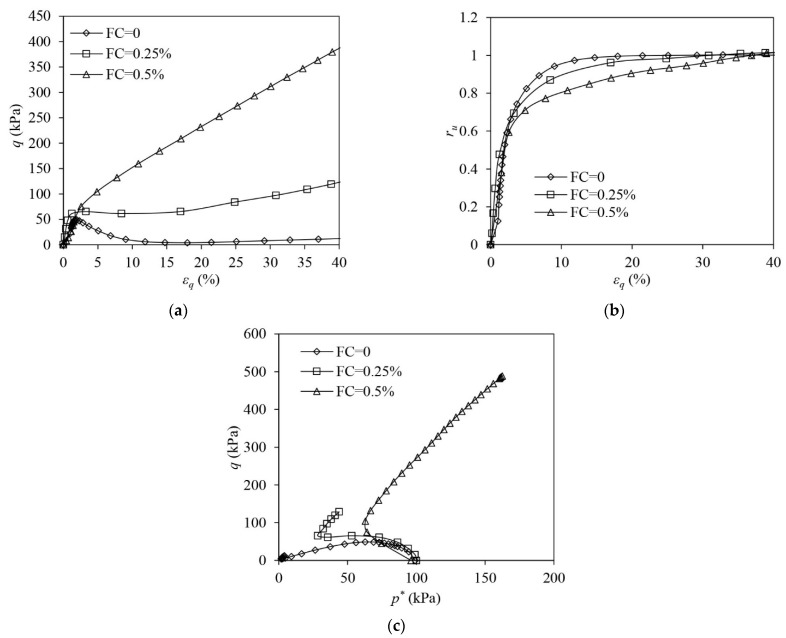
The test results of very loose samples under drained triaxial compression: (**a**) deviator stress–shear strain; (**b**) excess pore pressure ration–shear strain; (**c**) volumetric strain–shear strain.

The results of VL samples under undrained triaxial compression are shown in Figure 6. The unreinforced sample shows a typical static liquefaction response. The deviator stress increases at the very initial stage of the loading. The sample initiates instability once the deviator stress (q) approaches the peak. Thereafter, q drops significantly, which is accompanied by the development of flow deformation until the large shear strain (i.e., 40%) is attained, at which point the sample almost loses its undrained strength (Figure 6a). The EPP ratio ($r_{u}$) of the sample increases to unity and thus the effective stress of the sand skeleton drops to zero, indicating that a fluidised state occurs in the unreinforced sample (Figure 6b,c). Liquefaction becomes absent in the reinforced samples where q increases continuously with the development of $\varepsilon_{q}$ and never drops. When FC increases, the undrained strength of the sample becomes greater (Figure 6a). The reinforced samples show a tendency of suppressed contraction and the EPP continuously builds up, $r_{u}$ eventually approaching unity (Figure 6b). The ESP of the reinforced samples shifts leftwards first with the drop in $p^{*}$ due to the rise in the EPP at the initial loading stage. In the following stage, the ESPs reverse their directions to develop accompanied by the onset of the increase in $p^{*}$ (Figure 6c). Although $r_{u}$ = 1 is attained in the reinforced sample, the samples remain stable, and the liquefaction is stopped.

**Figure 7:**
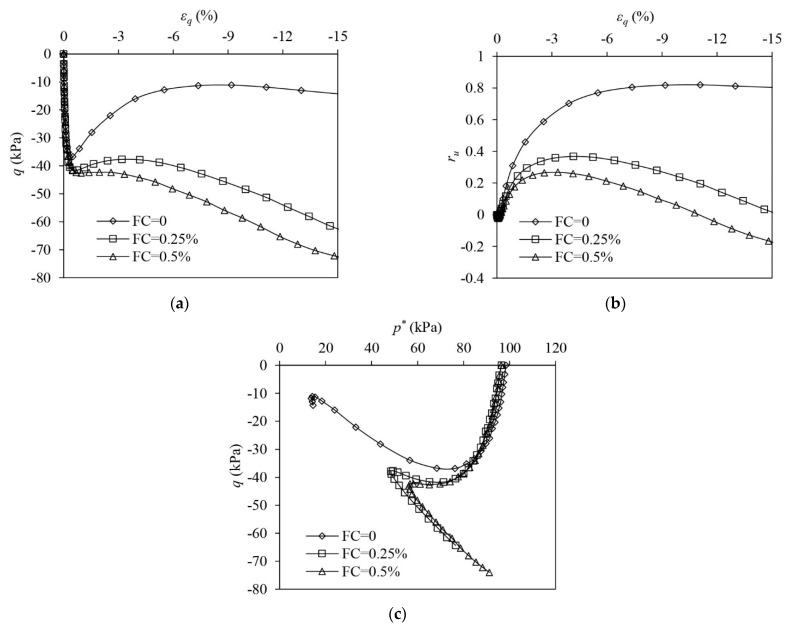
The test results of very loose samples under drained triaxial compression: (**a**) deviator stress–shear strain; (**b**) excess pore pressure ration–shear strain; (**c**) volumetric strain–shear strain.

Figure 7 shows the test results of VL samples under undrained triaxial extension. The unreinforced sample shows a strain-softening response involving a significant post-peak drop in q. q remains at a residual level and the sample never completely loses its undrained strength (Figure 7a). The unreinforced sample shows a continuous tendency of contraction. $r_{u}$ steadily increases and plateaus at around 0.82 (Figure 7b). The ESP of the unreinforced sand moves close to the origin of the $q:p^{\prime }$ plane, eventually attaining limited flow liquefaction (Figure 7c). The addition of fibres significantly suppresses the extent of strain-softening to develop in the sand. The reinforced samples show an initial softening, which is followed by a hardening (Figure 7a). The reinforced samples become less contractive than the unreinforced sample. After an initial growth, $r_{u}$ in the reinforced samples drops (Figure 7b). The ESP of the reinforced samples shifts leftwards due to the build-up in EPP first, and then the ESPs alter their direction to evolve because of the onset of phase transformation (PT). The PT may induce the response of the sample to switch to a tensioned dilation from an original tensioned contraction (Figure 7c) [47]. The occurrence of PT in the reinforced samples is also evidenced by the development of $r_{u}$ (Figure 7b). The $r_{u}$ increases first when the sample shows a tendency of contraction while $r_{u}$ drops once a tendency of dilation prevails. The reason for the ESP reversal of the reinforced samples is further discussed in the following section.

#### 3.2.2. L Samples

**Figure 8:**
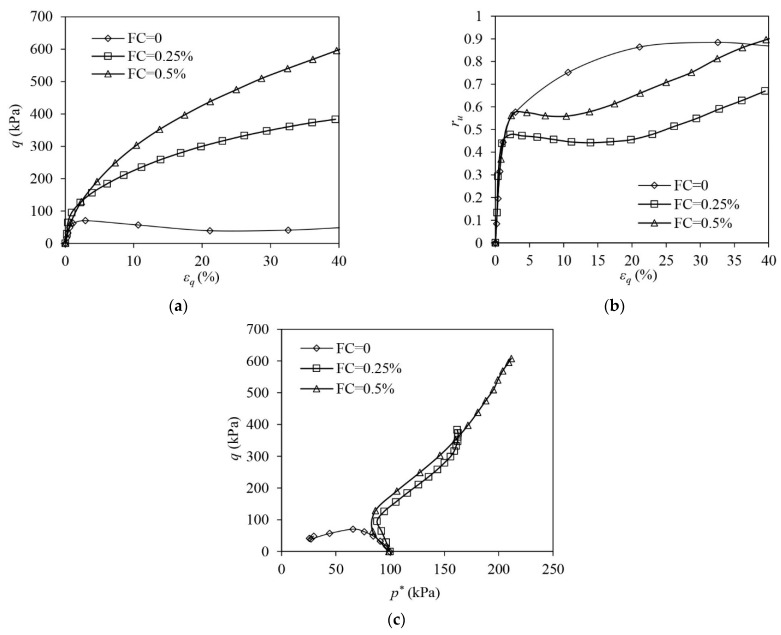
The test results of loose samples under undrained triaxial compression: (**a**) deviator stress–shear strain; (**b**) excess pore pressure ration–shear strain; (**c**) volumetric strain–shear strain.

Figure 8 illustrates the test results of the L samples under undrained triaxial compression. The unreinforced sample shows that limited flow liquefaction with strain softening occurs. A large EPP builds up in the unreinforced sample with $r_{u}$ reaching around 0.88. The unreinforced L sample shows a continuous tendency to contract until a large $\varepsilon_{q}$ of 40% has been attained. Similar to the VL samples (Figure 6), fibre reinforcement largely increases the undrained strength of L sand. When $\varepsilon_{q}$ = 40%, q of the reinforced sample with FC = 0.25% and 0.5%, respectively, increases by 691.2% and 1129.1% more than that of the unreinforced sample (Figure 8a). The presence of fibres stops the occurrence of strain softening. The EPP in the reinforced samples increases first and then drops slightly before it rises again at the large shear strains. It seems that the addition of fibres increases the dilation of the sample, which is evidenced by the lesser EPP build-up in the very initial stage of loading. However, with the increase in $\varepsilon_{q}$, the tensile stress in the fibres increases and the tendency of the sand skeleton to dilate is suppressed as greater tensile stress mobilised in the fibres imposes significant extra confining stress on the sand skeleton, which is further demonstrated in the following section (Figure 12). The samples show a tendency to contract afterwards, causing a rise in the EPP under large shear strains (Figure 8b). It also indicates that adding more fibres initiates the post-dilation contraction (PDC) more quickly. In the reinforced sample with FC = 0.5%, the PDC initiates at a $\varepsilon_{q}$ of around 10%, while in the reinforced sample with FC = 0.25%, the PDC starts at a $\varepsilon_{q}$ of around 15%. $r_{u}$ at $\varepsilon_{q}$ = 40% in the reinforced sample with FC = 0.5% is around 0.9, which is much greater than that in the reinforced sample with FC = 0.25% (i.e., 0.67). The ESP of the unreinforced sample continuously shifts leftwards with the build-up of the EPP, while the ESPs of the reinforced samples reverse the direction to evolve after initially moving leftwards (Figure 8c). The evolution of ESPs is even altered by the tensioned fibres under large shear strains, forcing the ESPs to slightly bend leftwards (Figure 8c). The PDC causes a rise in the positive EPP under large $\varepsilon_{q}$ in the reinforced samples (Figure 8b), which slightly reduces the effective mean stress of the composite (i.e., $p^{*}$), and thus the ESPs bend leftwards.

**Figure 9:**
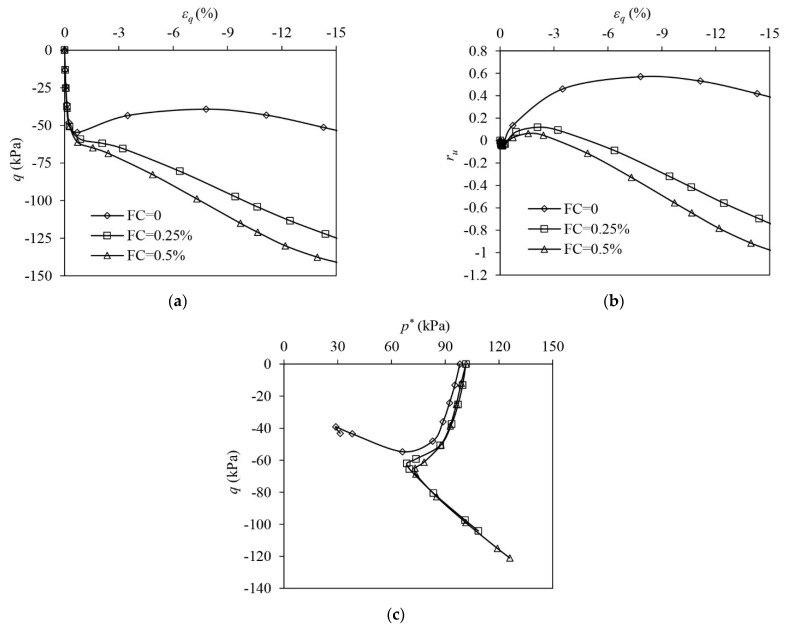
The test results of loose samples under undrained triaxial extension: (**a**) deviator stress–shear strain; (**b**) excess pore pressure ration–shear strain; (**c**) volumetric strain–shear strain.

The test results of L samples under undrained extension are shown in Figure 9. The unreinforced sample exhibits limited flow liquefaction with post-peak instability occurring, inducing the undrained strength to decrease from the peak to a quasi-steady level (Figure 9a). The EPP ratio ($r_{u}$) in the unreinforced sample increases and peaks at 0.57 (Figure 9b). The reinforced samples show strain-hardening responses and the q of the reinforced samples remains much larger than the unreinforced sample (Figure 9a). The reinforced samples show an initial tendency of contraction, which is followed by a significant tendency of dilation, evidenced by the significant drop in $r_{u}$ after the initial minor increase (Figure 9b). With the increase in FC, the PCD occurs at a slightly smaller $\varepsilon_{q}$, revealing that adding more fibres to the sample increases its tendency to dilation. From the ESPs of all samples, it can be seen that the unreinforced sample shows a contractive response while the reinforced samples show typical dilative responses (Figure 9c).

#### 3.2.3. MD Samples

**Figure 10:**
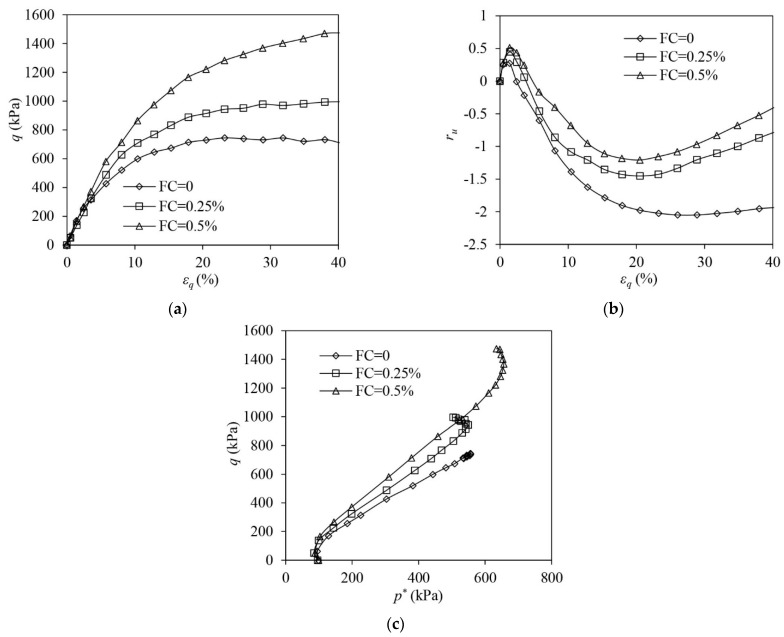
The test results of medium dense samples under undrained triaxial compression: (**a**) deviator stress–shear strain; (**b**) excess pore pressure ration–shear strain (**c**) volumetric strain–shear strain.

Figure 10 shows the test results of unreinforced and fibre-reinforced samples in MD states under undrained triaxial compression. All samples show strain-hardening responses. The presence of fibres significantly increases the undrained strength of the samples, and the strength improvement is boosted by the increase in FC (Figure 10a). $r_{u}$ increases at the very initial stage and thereafter it drops (Figure 10b). $r_{u}$ in the unreinforced sample drops to around −2, indicating the sample experiences a significant tendency to dilate. The presence of fibres suppresses the sand skeleton’s tendency of dilation when $\varepsilon_{q}$ becomes greater than 20%, and this suppression is strengthened by the increase in FC. With the following rise in $\varepsilon_{q}$, reinforced samples show a PDC and $r_{u}$ increases afterwards. The ESPs of the unreinforced and reinforced samples climb up along the linear paths with varied slopes, i.e., the stress ratios ($q/p^{\prime }$ or $q/p^{*}$), after the initial leftward shifting. With the increase in FC, the slope of the linear path followed by the ESP of the reinforced sample increases, as shown in Figure 10c. The suppressed contraction (the rise in EPP) under larger shear strains alters the ESPs of the reinforced samples. The ESPs deviate from the linear increase, shifting backwards due to the drop in $p^{*}$ (Figure 10c).

**Figure 11:**
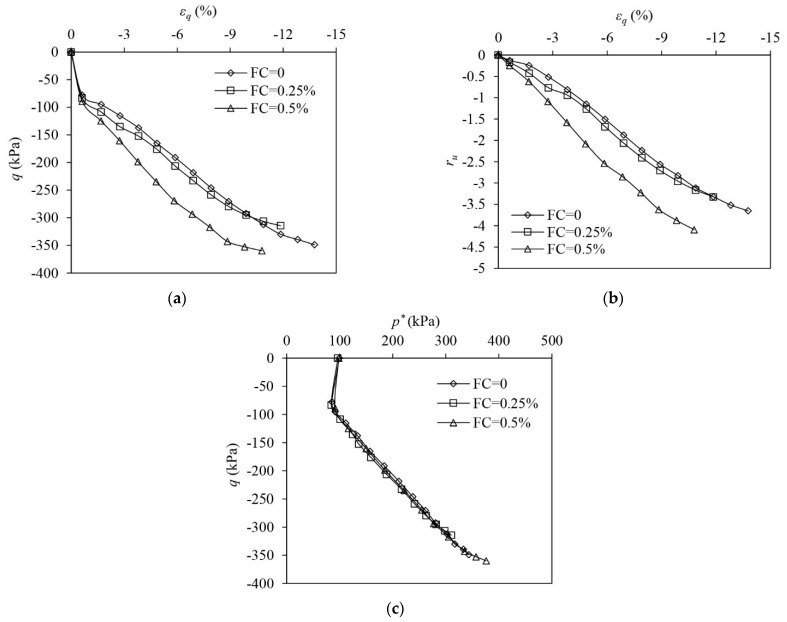
The test results of medium dense samples under undrained triaxial extension: (**a**) deviator stress–shear strain; (**b**) excess pore pressure ration–shear strain; (**c**) volumetric strain–shear strain.

Figure 11 shows the test results of unreinforced and fibre-reinforced samples in MD states under undrained triaxial extension. All samples roughly exhibit strain-hardening responses. However, the reinforced samples experience a slight drop in q when $\varepsilon_{q}$ ranges between 11 and 12%, which may be caused by sample necking occurring at a $\varepsilon_{q}$ of around −10%. The curves for the reinforced samples in Figure 11, therefore, stop once q approaches the peak. Adding fibres into MD sand roughly increases the undrained tensile strength of the sample (Figure 11a). All samples show tensioned dilation under the undrained extension. Fibre reinforcement increases the sand skeleton’s tendency of dilation, generating a greater negative EPP (Figure 11b). The ESPs of all samples show significant dilation, and the presence of fibres has an insignificant influence on the evolution of the ESP. After the initial reversal, the ESPs of unreinforced and fibre-reinforced samples evolve along a unique linear path (Figure 11c).

### 3.3. Effects Introduced by Fibre Reinforcement to Benefit the Liquefaction Resistance of Sand

**Figure 12:**
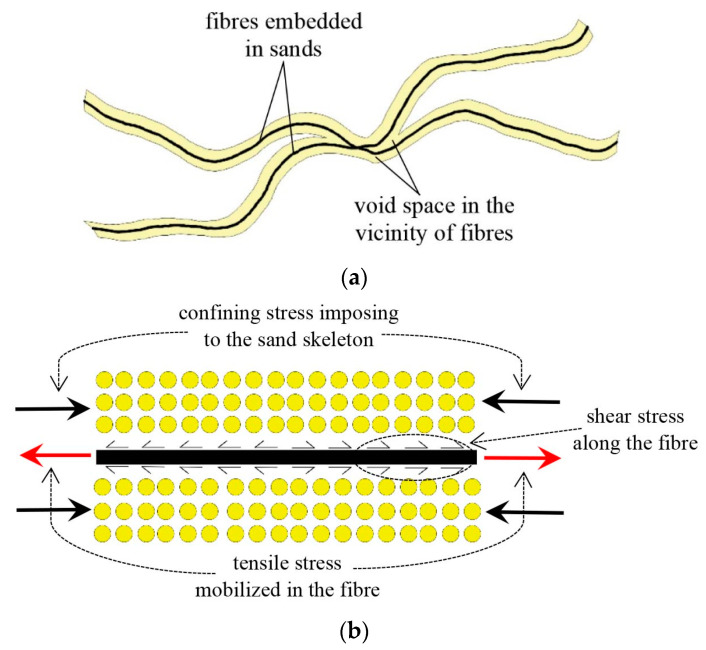
The illustration of the benefiting effects of fibres: (**a**) densifying effect and (**b**) confining effect.

Fibre reinforcement may introduce two main effects, i.e., densifying effect and confining effect, to benefit the liquefaction resistance of sand. Fibres densify the sand skeleton by occupying partial voids in the composite. Fibres also take up extra space in the vicinity around the embedded fibres (i.e., perturbed zone) to form fibre space, which may cause further densification of the sand skeleton [9,20,48,49] (Figure 12a). The densifying effect caused by fibres may increase the sand skeleton’s tendency of dilation. As a result, the build-up of the positive EPP is suppressed and the liquefaction resistance of the sand is improved.

Fibres are tension-resistant and seen as a reactive component in reinforced sand. Tensile stress mobilised in tensioned fibres may apply extra confining stress to the sand skeleton through the interaction between the fibres and sand skeleton, as illustrated in Figure 12b, which is the so-called confining effect here. The confining effect may stabilise the samples and prevent a liquefied state from developing after $r_{u}$ = 1 is attained [31]. However, the confining effect may highly depend on the extent to which the fibres are tensioned.

Both the densifying effect and confining effect caused by the fibres improve the liquefaction resistance of the sand. The two effects mentioned above may interplay with each other and the improvement in liquefaction resistance may be determined by their coupling effects. For example, the confining effect may significantly curb the exertion of the densifying effect, as observed in Figure 8b and Figure 10b.

### 3.4. The Characteristic Lines

#### 3.4.1. The Strength Envelope

**Figure 13:**
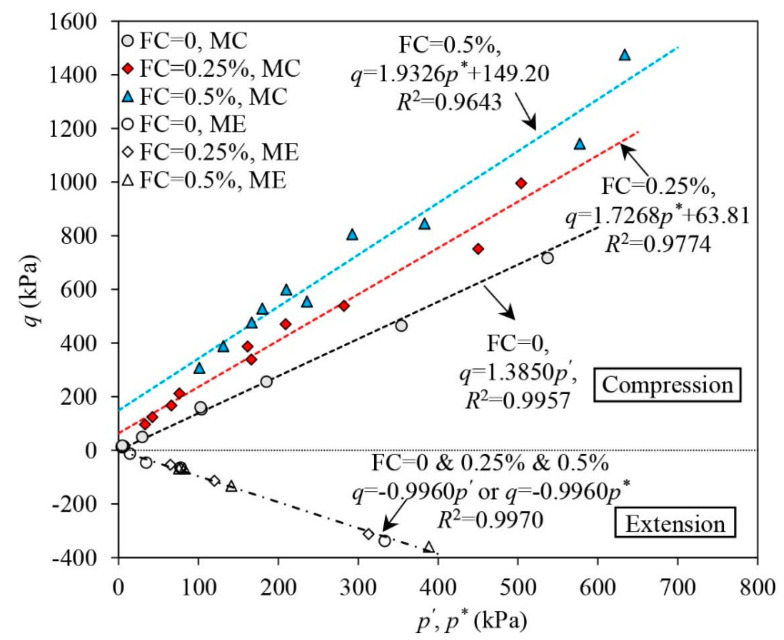
The strength envelops of the samples.

Figure 13 shows the strength envelopes of both unreinforced and fibre-reinforced sand. The strength envelope was determined by the effective stress states of the samples when $\varepsilon_{q}$ approaches 40% under triaxial compression while −12% under triaxial extension. Under triaxial extension, the strength envelope was determined at $\varepsilon_{q}$ = −12%, as necking of the samples initiates when $\varepsilon_{q}$ ranges between −10% and −12%. In some cases, the deviator load of the sample dropped significantly once $\varepsilon_{q}$ became greater than 12% due to sample necking.

The effects of the fibre reinforcement on the strength envelope are varied under triaxial compression and extension. The addition of fibres considerably changes the strength envelope under triaxial compression while the strength envelope under triaxial extension is not largely affected by fibres. Under triaxial compression, the strength envelope of the unreinforced sand is a straight line radiating from the origin of the $q:p^{\prime }$ plane. The strength envelope of the unreinforced sand is also referred to as the critical state line (CSL), which determines the stress ratio of the sand skeleton when the critical state is approached under large shear strains (i.e., 40%). The strength envelope of the unreinforced sand can be well-fitted using a linear regression function, $q=1.3850p^{\prime }$, yielding a regression coefficient (i.e., *R*^2^) of 0.9957. Adding fibres alters the strength envelope. With the increase in FC, both the intercept and the slope of the strength envelope increase. When FC = 0.25% and 0.5%, the strength envelopes can be, respectively, well fitted using linear regression functions of $q=1.7268p^{*}+63.81$ and $q=1.9326p^{*}+149.20$. The regression coefficients for FC = 0.25% and 0.5% are, respectively, 0.9774 and 0.9643. The presence of fibres seems to make the cohesionless sand have a “fake cohesion” and thus overcome the sand’s fragility. Under triaxial extension, the effect of the fibre reinforcement on the strength envelope is ignorable. A straight line passing through the origin of the stress plane $\left(q:p^{\prime }\;or\;q:p^{*}\right)$ fits the stress data at $\varepsilon_{q}$ = −12% well, indicating the fibres are incapable of improving the strength response of sand under triaxial extension.

#### 3.4.2. The ESP Reversal Line

Under undrained triaxial loading, the ESP of the medium dense to dense sand may alter its direction to shift (ESP reversal), at which the change of the mean effective stress alters from an initial decrease to a following increase, indicating the response of the sand converts from a suppressed contraction to a suppressed dilation, which is also referred to as phase transformation (PT) [47,50]. The ESP reversal is also experienced by the reinforced samples even though they are in VL and L states (Figure 6, Figure 7, Figure 8 and Figure 9). As discussed in Section 3.3, both the densifying and confining effects may cause ESP reversal. The densifying effect increases the tendency to dilate in sand and therefore forces the ESP to change its direction to shift due to the possible PT. Tensioned fibres contribute both deviator stress and mean stress to the composite and thus change the evolution of ESP of reinforced sand. The stress contribution of fibres is further discussed in the following section.

**Figure 14:**
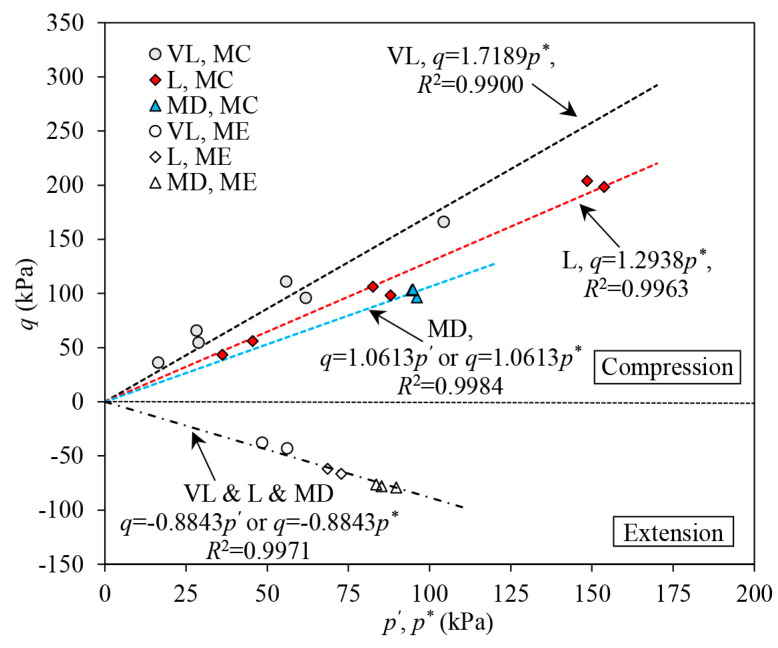
The ESP reversal line of samples.

Figure 14 shows the data points at which the ESPs of samples alter the direction to shift. For the unreinforced samples in the VL and L states, suppressed contraction predominantly occurs, and thus only the data from the reinforced samples are included. For MD samples, both the data points for unreinforced and reinforced samples are contained.

Under triaxial compression, the data collected from the samples in different density states can be well-fitted with straight lines passing through the origin. The slope of the fitted ESP reversal line drops when the samples are in a denser state, as shown in Figure 14. In the VL and L samples, the stress ratio (i.e., $q/p^{*})$ at which the ESP reversal occurs remains indifferent to the FC and the confining stress. In MD samples, adding fibres does not significantly affect the PT characteristics of the sand skeleton. The stress ratio initiating the PT in the unreinforced sand remains quite close to that causing the ESP reversal in the reinforced sample. Under triaxial extension, the stress ratios leading to the reversal in ESPs remain consistent across different samples. The data for both the unreinforced sample in MD state and the reinforced samples in VL, L and MD states roughly fall on a straight line going through the origin of the stress plane (Figure 14).

#### 3.4.3. The Mechanism Correlating to the Strength Envelope and ESP Reversal of the Fibre Reinforced Sand

##### Strength Envelope

To discuss the mechanism correlating the ESP reversal and strength envelope of the fibre-reinforced sand, the constitutive model based on the rule of the mixture is introduced [39] (Equation (3)). The model assumes that each component in the fibre-reinforced composite obeys its own constitutive laws. The effective stresses acting on the composite are superpositions of the stress contributions of fibres and the sand skeleton after scaling using their volumetric concentrations as per the rule of mixtures.

(3) $$\left[\begin{array}{c} \dot{p^{*}}\\ \dot{q^{*}} \end{array}\right]=\mu_{m}\left[\begin{array}{c} \dot{p^{\prime }}\\ \dot{q^{\prime }} \end{array}\right]+\mu_{f}\left[\begin{array}{c} \dot{p_{f}}\\ \dot{q_{f}} \end{array}\right]$$

In Equation (3), $\mu_{m}$ and $\mu_{f}$, respectively, represent the volumetric concentration of the sand skeleton and the fibres. $\mu_{m}$ = (*V*_s_ + *V*_v_)/*V* = (*V* + *V_f_*)/*V* while $\mu_{f}$ = *V_f_*/*V*. Here *V*_s_, *V*_v_, *V_f_* and *V*, respectively, represent the volume of the sand skeleton, the volume of the voids, the volume of the fibres and the volume of the overall composite. Considering *V_f_* is far less than *V*, it is rational to presume $\mu_{m}$ = 1.

**Figure 15:**
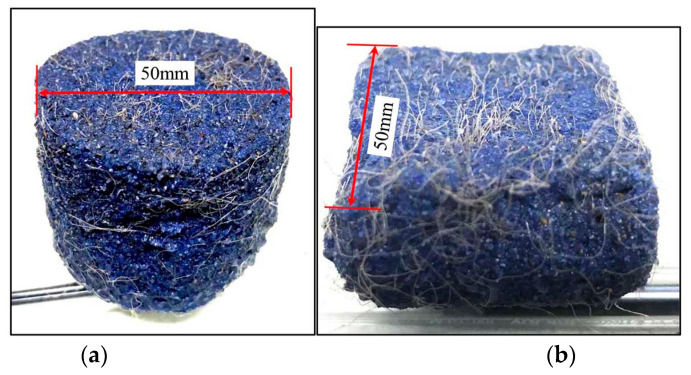
Sectioned frozen fibre-reinforced sample with FC = 0.15%: (**a**) horizontal section; (**b**) vertical section.

The stress contributions of fibres to the host soils may be largely affected by the distributions of fibre orientations and the loading paths, as fibres are usually unidirectionally tension-resistant. Following the procedures proposed by Diambra [37], the orientation distribution of fibres was investigated. The saturated fibre-reinforced samples were made using the moist tamping method and then frozen together with the mould. Thereafter, the sample was cut horizontally and vertically (Figure 15). The number of fibres intersecting finite areas of 20 mm × 20 mm in the horizontal (*N*_H_) and vertical (*N*_V_) sections were counted. To facilitate the counting work, the sand was dyed blue to make a good contrast to the fibres (Figure 15). After the counting work on four different samples, it was found that the average ratio of *N*_V_/*N*_H_ was 2.05, indicating fibres are more prone to orientating horizontally. By adopting a spherical coordinate, a function ρ(θ), representing the volumetric concentration of fibres distributed in an infinitesimal volume lying with an angle θ off the horizontal plane, can describe the distribution of fibre orientations in the fibre-reinforced sand [36,37]. With the counting results, Equation (4) yielded a good description of the fibre orientation distribution.

(4) $$\rho (\theta )=2.04\mu_{f}|{cos}^{5}\theta |$$

**Figure 16:**
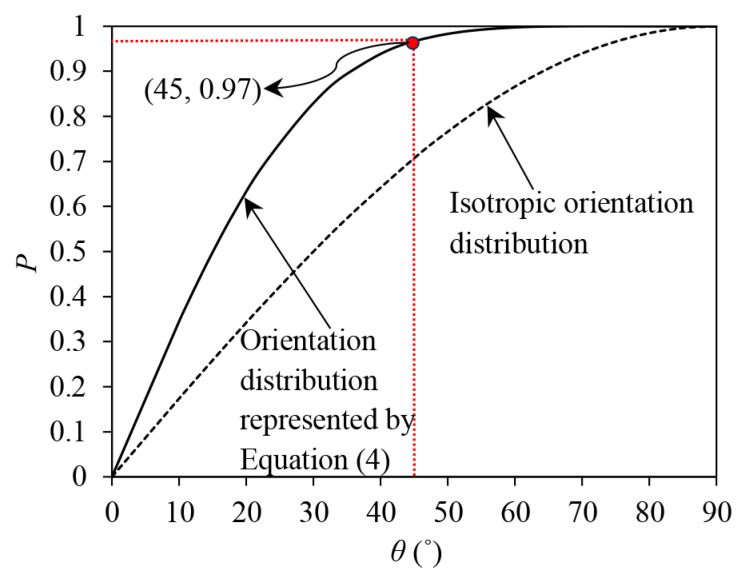
The ratio (*P*) of fibres orientated ±θ from the horizontal over the total volume of the fibres.

Equation (4) represents a fibre orientation distribution in which 97% of fibres are orientated ±π/4 from the horizontal, as demonstrated in Figure 16. In Figure 16, the isotropic distribution of fibre orientation, which can be described by ρ(θ) = $\mu_{f}$, is also plotted to show a comparison. Considering the significant sub-horizontal orientation distribution of fibres, the fibres are more likely to be tensioned under triaxial compression and thus more stress contributions may be made by fibres, while under triaxial extension, fibres are tensioned less and the stress contribution may be insignificant.

The constitutive model (Equation (3)) reveals that the strength envelope of the reinforced sand is controlled by both the stress contributions of the fibres and sand skeleton. When the sand skeleton approaches the critical state, the strength envelope of the composite may be determined after shifting the CSL of the sand skeleton upward by the amount representing the stress contribution of the fibres.

Under triaxial compression, the fibres are significantly tensioned, and considerable stress contributions are made by the fibres, leading to the stress envelope of reinforced sand deviating from the CSL of the sand skeleton. The stress contributions of the fibres are ignorable under triaxial extension, and thus the strength envelope of the reinforced sand remains roughly consistent with the sand skeleton. What should be mentioned is that under triaxial compression, adding fibres causes certain “fake cohesion” to the sand, meaning that effective interaction between the fibres and sand skeleton may prevail even though $p^{*}$ drops to zero, as discussed by Diambra and Ibraim [51] and Zhang and Russell [29].

##### ESP Reversal

**Figure 17:**
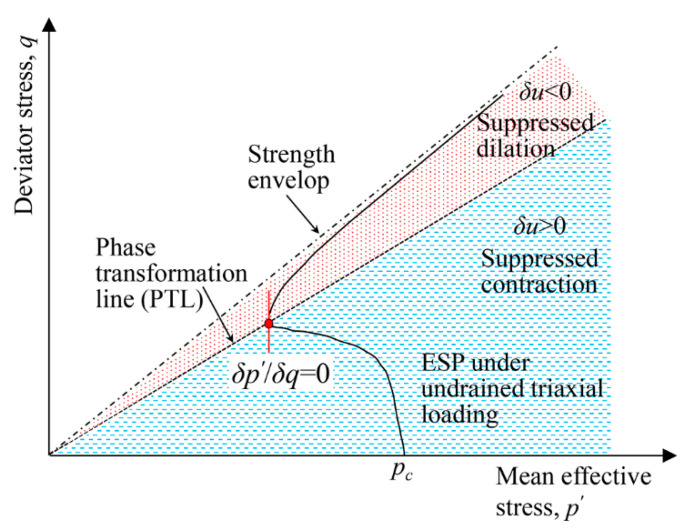
The illustration of the phase transformation line (PTL) and the definition of the phase transformation point in unreinforced sand.

Figure 17 demonstrates the phase transformation line (PTL) of the unreinforced sand under undrained triaxial loading as well as the definition of the PT point. From the aspect of strain, the PT corresponds to the moment at which the tendency of the volumetric change alters from suppressed contraction to suppressed dilation. The increment of the EPP (i.e., u), correspondingly, switches from δu > 0 to δu < 0, and thus δu = 0 is attained at the PT point [47,50]. However, from the aspect of stress, $\delta p^{\prime }/\delta q$ = 0 is achieved at the PT point [30] (Figure 17). The PT point defined from the stress and strain aspects may not completely coincide with each other. Considering the effective stress principle, i.e., $p^{\prime }$ = p−u, $\delta p^{\prime }$ = 0 is equivalent to δp − δu = 0, which may be attained slightly earlier than δu = 0.

For the reinforced sand subjected to undrained triaxial loading, ESP reversal occurs when

(5) $$\frac{\delta p^{*}}{\delta q^{*}}=0$$

Following the constitutive model and considering that $\delta q^{*}$ > 0 at the ESP reversal moment, ESP reversal of the reinforced samples occurs when Equation (6) is workable.

(6) $$\delta p^{*}=\delta \mu_{f}p_{f}+\delta p^{\prime }=0$$

Equation (6) indicates that the ESP reversal in the reinforced sand depends on both the increment in effective mean stress contribution of the fibres (i.e., $\delta \mu_{f}p_{f}$) and the increment in effective mean stress of the sand skeleton (i.e., $\delta p^{\prime }$).

After introducing the relationship between total mean stress and effective mean stress in the fibre-reinforced sand, i.e., $p^{*}=p-u$, into Equation (3), it gives:

(7) $$\delta p-\delta p^{\prime }=\delta \mu_{f}p_{f}+\delta u$$

According to the two definitions of PT point form aspects of stress and strain, it may be inferred that in the reinforced sand, ESP reversal of the sand skeleton may occur when $\delta \mu_{f}p_{f}+\delta u$ = 0. Here, ${\delta \mu }_{f}p_{f}+\delta u$ represents the increment in the overall magnitude of EPP being ‘felt’ by the sand skeleton during undrained loading.

Zhang and Russell [31] discussed the irrationality of using the conventional EPP ratio (i.e., $r_{u}$) to assess the liquefaction resistance of fibre-reinforced sand. An alternative EPP ratio, denoted as $r_{u}^{*}$, was proposed to provide a better indication of the liquefaction in the reinforced sand, as given in Equation (8). When $r_{u}^{*}$ = 1, the effective mean stress of the sand skeleton ($p^{\prime }$) decreases to zero and a liquefied state is attained in the fibre-reinforced sand.

(8) $$r_{u}^{*}=\frac{u+\mu_{f}p_{f}}{p}$$

**Figure 18:**
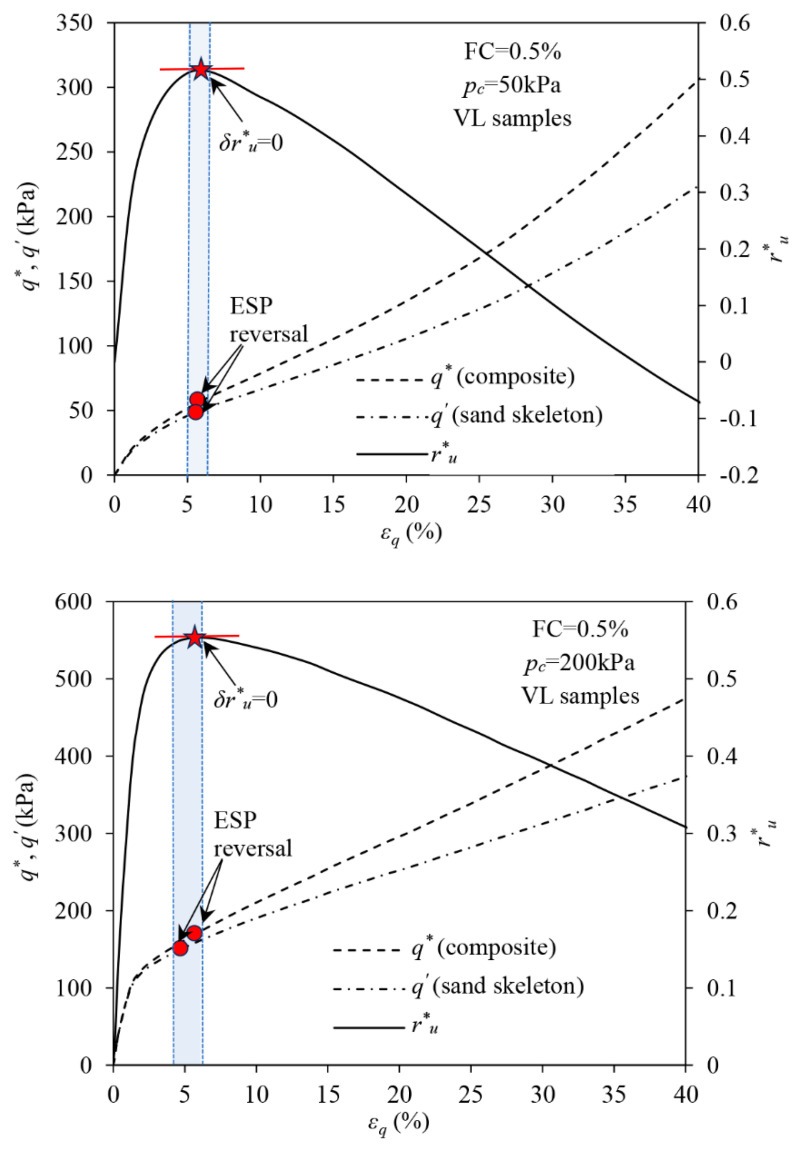
The link between the initiation of ESP reversal in the fibre-reinforced sand and the attainment of ${\delta r}_{u}^{*}$= 0.

Following the discussion above, the ESP reversal of the sand skeleton in the reinforced sand may initiate when the increment in the alternative EPP ratio equals zero, i.e., ${\delta r}_{u}^{*}$ = 0. Figure 18 shows the evolution of the stress–strain curves of the sand skeleton, composite as well as the evolution in $r_{u}^{*}$ in the reinforced sand in VL states; the details of $r_{u}^{*}$ can be found in Zhang and Russell [31]. The ESP reversal points and the peaks of the $r_{u}^{*}$ at which ${\delta r}_{u}^{*}$ = 0 are also labelled with a red circle and a red star. It can be seen that the ESP reversal points of the sand skeleton and the composite are quite close to each other. The ESP reversal also occurs at a moment quite close to ${\delta r}_{u}^{*}$ = 0.

When ${\delta r}_{u}^{*}$ = 0, $\delta \mu_{f}p_{f}+\delta u$ = 0 also prevails. In a denser sample, a greater tendency to dilate may suppress the generation of the positive EPP. Therefore, lesser mean stress contribution of fibres may be required to balance out the EPP. The ESP reversal in a denser sample thus occurs at a lower stress ratio.

## 4. Discussion on the Anisotropy in the Liquefaction Resistance of the Fibre-Reinforced Sand

### 4.1. The Stress Contribution of Fibres

**Figure 19:**
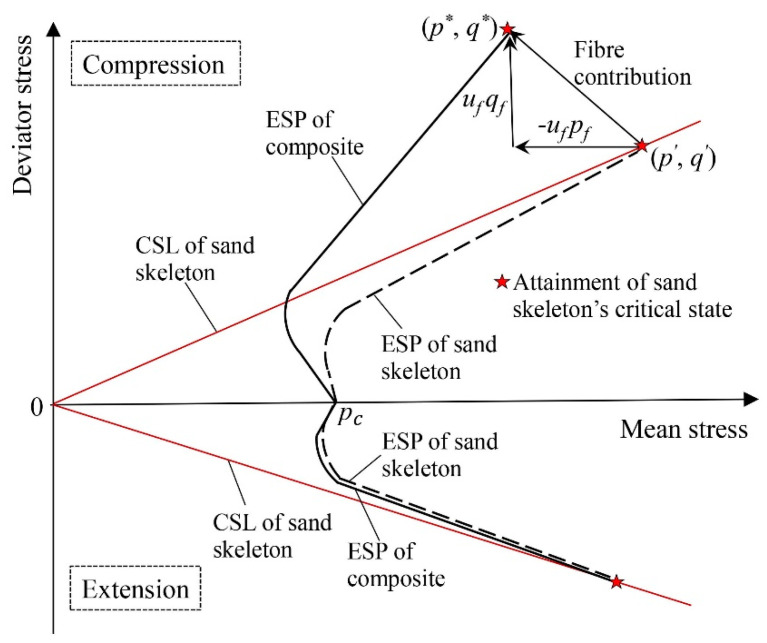
The illustration of the fibre stress contribution under undrained triaxial compression and extension.

In the reinforced sample, the tensioned fibres contribute stresses to the composite and thus separate the ESPs of the composite and the sand skeleton during undrained triaxial loading (Figure 19). Once significant fibre stress contribution prevails, the ESP of the composite may greatly exceed the CSL of the sand skeleton (i.e., compression in Figure 19). However, when the stress contribution of the fibres is insignificant, the ESPs of the composite and the sand skeleton may become quite close to each other (i.e., extension in Figure 19). In this study, the fibre stress contribution was estimated by introducing the constitutive modelling framework based on the rule of mixture [39], as given in Equation (3).

The model assumes that the fibres behave elastically while the strains of the fibres are incompatible with the composite. When a dimensionless factor is introduced to link the strains of the fibres and the composite, the stress contribution of fibres can be written as Equation (9).

(9) $$\mu_{f}\left[\begin{array}{c} \dot{p_{f}}\\ \dot{q_{f}} \end{array}\right]=E_{f}f_{b}\left[\begin{array}{cc} M_{11} & M_{12}\\ M_{21} & M_{22} \end{array}\right]\left[\begin{array}{c} \dot{\varepsilon_{v}}\\ \dot{\varepsilon_{q}} \end{array}\right]$$

(10) $$\left[\begin{array}{l} M_{11}\\ M_{12}\\ M_{21}\\ M_{22} \end{array}\right]=\left[\begin{array}{cccc} 1/9 & 1/9 & 2/9 & 2/9\\ 1/3 & -1/6 & 2/3 & -1/3\\ 1/3 & 1/3 & -1/3 & -1/3\\ 1 & -1/2 & -1 & 1/2 \end{array}\right]\left[\begin{array}{l} \int_{\beta_{1}}^{\beta_{2}}\rho (\theta )\cos(\theta )\sin^{4}(\theta )d\theta \\ \int_{\beta_{1}}^{\beta_{2}}\rho (\theta )\cos^{3}(\theta )\sin^{2}(\theta )d\theta \\ 1/2\int_{\beta_{1}}^{\beta_{2}}\rho (\theta )\cos^{3}(\theta )\sin^{2}(\theta )d\theta \\ 1/2\int_{\beta_{1}}^{\beta_{2}}\rho (\theta )\cos^{5}(\theta )d\theta \end{array}\right]$$

Under undrained triaxial loading, the volumetric strain of the sample is always zero, and thus Equation (9) can be simplified to:

(11) $$\mu_{f}\left[\begin{array}{c} \dot{p_{f}}\\ \dot{q_{f}} \end{array}\right]=E_{f}f_{b}\left[\begin{array}{c} M_{12}\\ M_{22} \end{array}\right]\dot{\varepsilon_{q}}$$

In Equation (9), $E_{f}$ is the elastic modulus of fibres. Here, $E_{f}$ = 900 MPa, which was determined by conducting tension tests on a single Loksand fibre [39]. Considering that fibres may slide through the sand skeleton when the sample deforms, $f_{b}$, a dimensionless sliding factor, is introduced to quantify the relative sliding between fibres and the sand skeleton. Generally, an imperfect bond prevails between fibres and the sand skeleton and full sliding is also absent, leading to the magnitude of $f_{b}$ ranging from 0 to 1. $f_{b}$ = 0 and 1, respectively, represent a complete sliding and a perfect bond. It was found that the magnitude of $f_{b}$ is closely correlated to the mean effective stress acting on the fibre–sand composite (i.e., $p^{*})$, and $f_{b}$ can be estimated using a function of $p^{*}$, i.e., $f_{b}$ = $f(p^{*})$ [31,39]. In this study, only the stress contribution of fibres at a shear strain of 40% (under triaxial compression) was calculated by solving the equation set (Equation (12)) where the detailed $f_{b}$ is not required.

In Equation (10), $\beta_{1}$ and $\beta_{2}$ represent the integration limits, ensuring that when calculating the stress contributions of fibres, only the fibres that act in tension carry the stresses imposed on the composite. Under the triaxial compression loading condition, $\beta_{1}$ = 0 and $\beta_{2}$ = arctan $\sqrt{-\dot{\varepsilon_{r}}/\dot{\varepsilon_{a}}}$, while under the triaxial extension loading condition, $\beta_{1}$= arctan $\sqrt{-\dot{\varepsilon_{r}}/\dot{\varepsilon_{a}}}$ and $\beta_{2}$ = π/2. Here, $\dot{\varepsilon_{r}}$ and $\dot{\varepsilon_{a}}$ are, respectively, the incremental of the radial strain and the axial strain in the triaxial tests. ρ(θ) in Equation (3) is the function that describes the distribution of fibre orientations. The details of ρ(θ) are given in Equation (4).

When calculating the fibre stress, it was assumed that the sand skeleton approaches the critical state at $\varepsilon_{q}$ = 40% (under triaxial compression) and $\varepsilon_{q}$ = −12% (under triaxial extension). However, it was observed that under triaxial extension, the strength envelope of the fibre–sand composite remains the same as that of the sand skeleton (Figure 13), indicating the stress contribution of the fibres is ignorable. It is therefore assumed that the liquefaction resistance of sand under undrained triaxial extension is not largely affected by the confining effect. The stress contribution of fibres at the $\varepsilon_{q}$ = 40% under triaxial compression was then estimated by solving the following equation set (Equation (12)):

(12) $$\left\{\begin{array}{l} q^{*}-\mu_{f}q_{f}=q^{\prime }\\ p^{*}-\mu_{f}p_{f}=p^{\prime }\\ q^{\prime }=M_{cs}p^{\prime }\\ \mu_{f}p_{f}=\frac{M_{12}}{M_{22}}\mu_{f}q_{f} \end{array}\right.$$

In Equation (12), $M_{cs}$ represents the stress ratio of the sand skeleton at the critical state, here $M_{cs}$ = 1.39. $M_{12}$ and $M_{22}$ are the two terms in the stiffness matrix in the constitutive model, as shown in Equation (10). In the undrained loading, $M_{12}$ and $M_{22}$ are constants, yielding a constant ratio of $M_{12}$/$M_{22}$ = −0.7887. In Equation (12), $p^{*}$ and $q^{*}$ can be obtained from the test results, while $p^{\prime }$, $q^{\prime }$, $\mu_{f}p_{f}$ and $\mu_{f}q_{f}$ are unknown and can be obtained by solving Equation (12).

**Figure 20:**
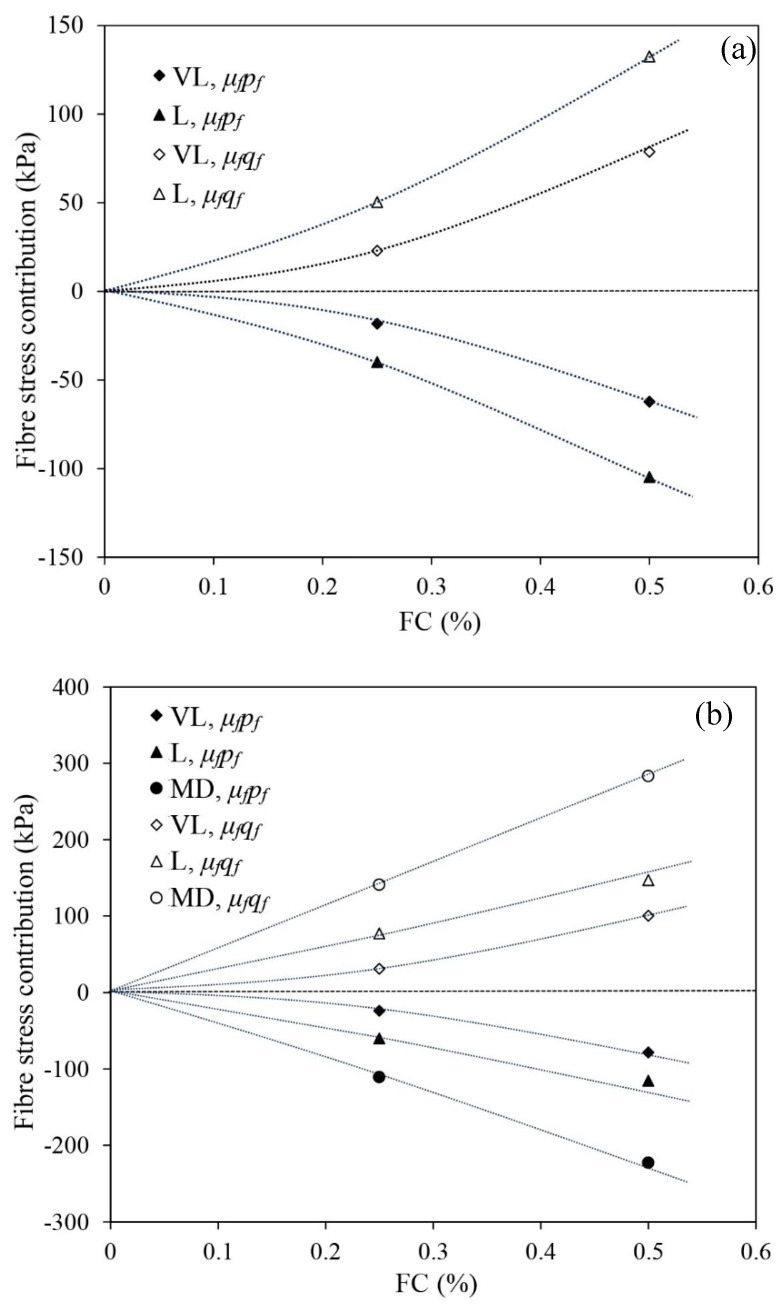

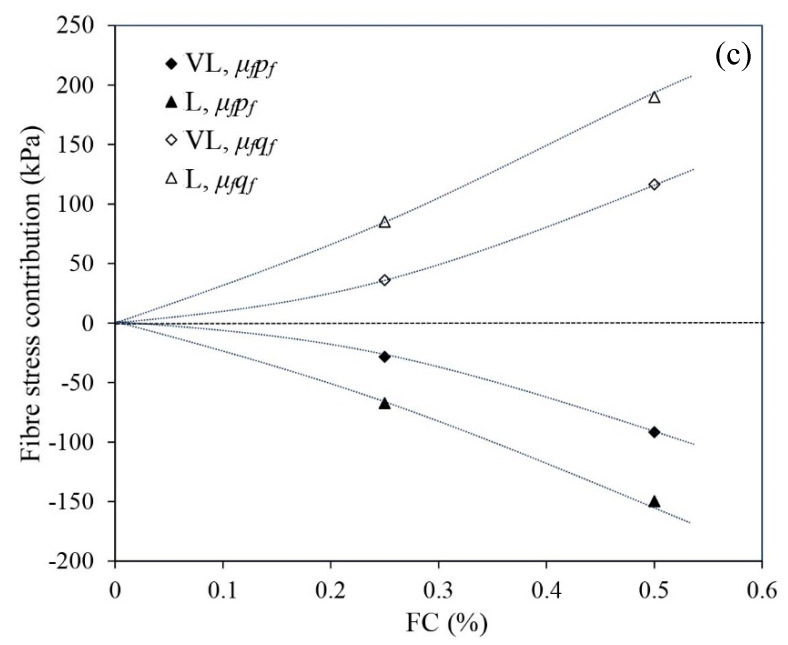
The calculated stress contributions of fibres at $\varepsilon_{q}$ = 40% under undrained triaxial compression: (**a**) *p_c_* = 50 kPa, (**b**) *p_c_* = 100 kPa and (**c**) *p_c_* = 200 kPa.

Figure 20 shows the fibre stress contribution under undrained triaxial compression. Fibres are unidirectional tension-resistant elements, and they only carry tensile load mobilised by the tensile strain of the sample. Fibres are more prone to being tensioned and contribute more stress to the composite under triaxial compression, as the maximum tensile strain of the sample (composite) is horizontal and most fibres have near-horizontal orientations in the samples produced using moist tamping [31,39]. It follows that the stress contribution of fibres in both horizontal ($\mu_{f}\sigma_{rf}$) and vertical ($\mu_{f}\sigma_{af}$) directions are tensile (negative) stress, while $\mu_{f}\sigma_{rf}$ has a greater magnitude than $\mu_{f}\sigma_{af}$. As a consequence, under undrained triaxial compression, the mean stress contribution of fibres ($\mu_{f}p_{f}$) is negative, representing the amount by which $p^{\prime }$ shifts rightward from $p^{*}$. The deviator stress contribution of fibres ($\mu_{f}q_{f}$) is positive, representing the amount by which $q^{\prime }$ shifts downwards from $q^{*}$.

Figure 20 indicates that under triaxial compression, the fibres make significant stress contributions. With the rise in FC, the stress contribution increases. The fibre stress contribution also increases when the reinforced samples are in a denser state, as a better interaction may prevail between the fibres and sand skeleton. A greater confining stress also provides a more optimal interaction between the fibre and skeleton, as the interlock between the fibres and sand skeleton is somehow dependent on the effective mean stress acting on the composite (i.e., $p^{*}$), yielding a greater fibre stress contribution under greater confining stresses.

### 4.2. The Liquefaction Resistance

**Figure 21:**
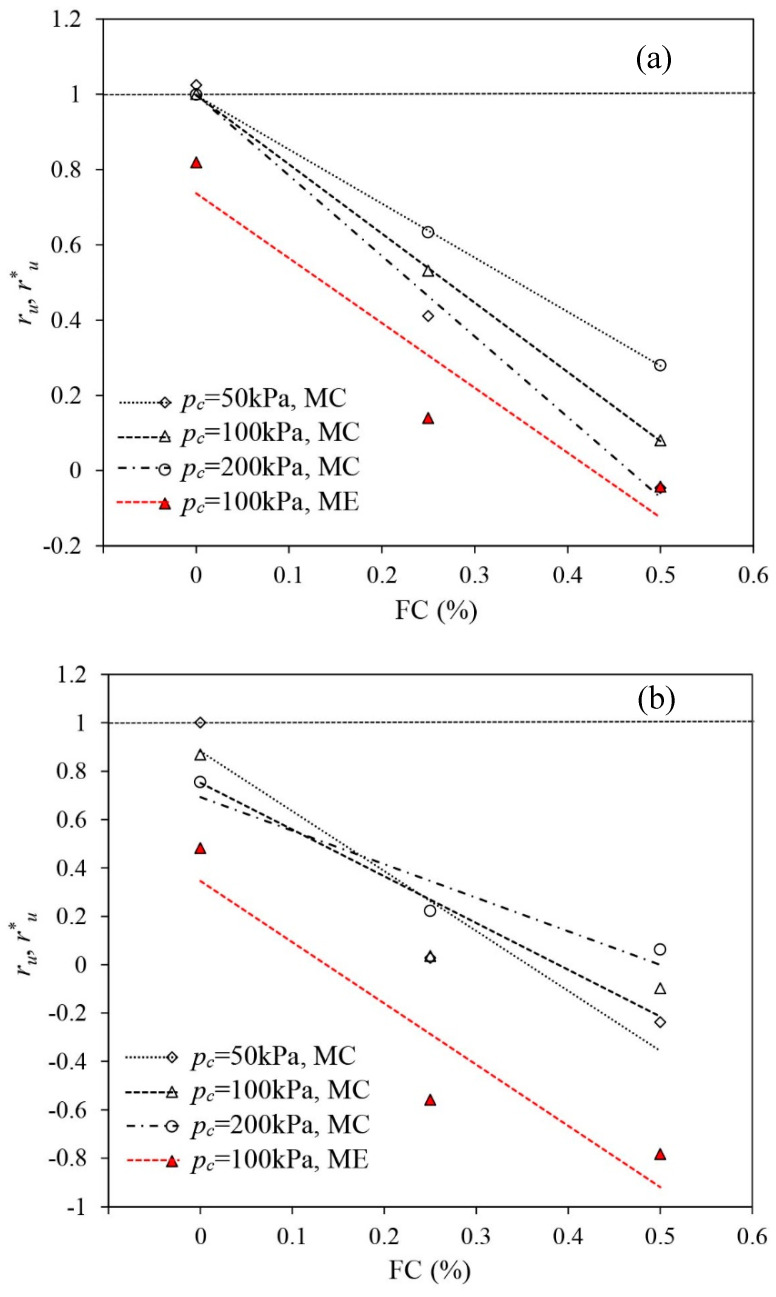

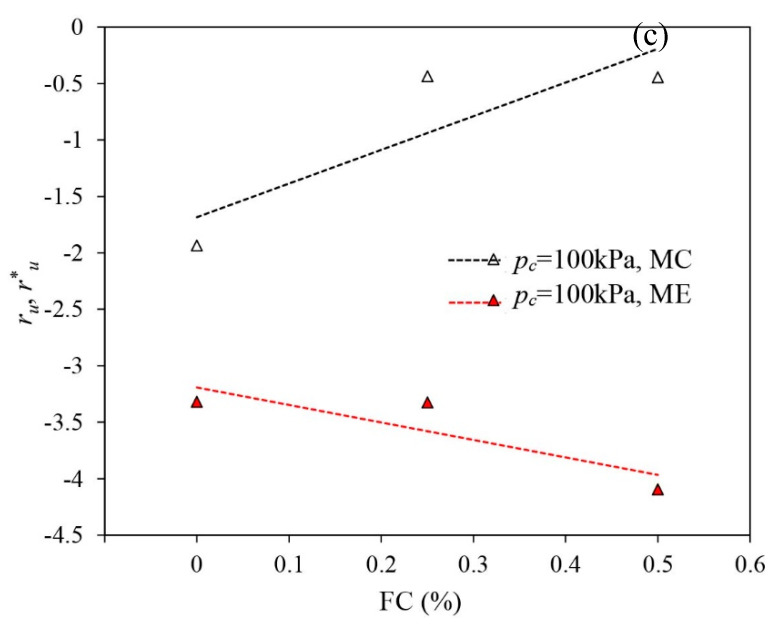
The liquefaction state indicated by the EPP ratios of $r_{u}$ and $r_{u}^{*}$ of all samples: (**a**) VL samples, (**b**) L samples and (**c**) MD samples.

To illustrate the final liquefaction states of all samples, e.g., at $\varepsilon_{q}$ = 40% under triaxial compression while at $\varepsilon_{q}$ = −12% under triaxial extension, $r_{u}^{*}$ of the reinforced samples under triaxial compression and $r_{u}$ of the unreinforced samples as well as the reinforced samples under triaxial extension are shown in Figure 21.

In VL states, the unreinforced samples are susceptible to liquefaction. Under undrained triaxial compression, $r_{u}$ in all unreinforced samples subjected to different confining stresses increases to unity and liquefaction is attained. The effective stress of the unreinforced samples drops to zero and the samples almost completely lose their undrained strength. Under the undrained triaxial extension, the unreinforced sample shows a significant strain-softening response and $r_{u}$ approaches around 0.82. The sample largely loses its undrained strength, and a limited flow liquefaction occurs. Under undrained triaxial compression, adding fibres significantly decreases $r_{u}^{*}$, being much lower than 1, meaning that the reinforced samples remain far from a liquefied state. Adding more fibres makes $r_{u}^{*}$ drop more. The reduction in $r_{u}^{*}$ predominantly benefits from the stress contribution of the fibres (i.e., confining effect). Under the undrained triaxial extension, adding fibres also makes $r_{u}$ decrease and a liquefied state is absent in the reinforced samples. However, under the undrained triaxial extension, the reduction in $r_{u}$ is mainly caused by the added dilating tendency benefiting from the densifying effect.

When unreinforced samples in L states are under undrained triaxial compression, significant strain softening occurs irrespective of the confining stress. $r_{u}$ at $\varepsilon_{q}$ = 40% in the unreinforced sand decreases with the growth in confining stress. When $p_{c}$ = 50 kPa, the sample becomes liquefied with $r_{u}$ approaching unity. However, when $p_{c}$ = 100 kPa and 200 kPa, limited flow liquefaction occurs in the unreinforced samples instead. In comparison with $p_{c}=$ 50 kPa, the lesser liquefaction potential of unreinforced samples under $p_{c}$ = 100 kPa and 200 kPa may result from the greater densification experienced by the samples during the consolidation stage. Similar to the observations in the VL samples, fibre reinforcement significantly suppresses the liquefaction potential of L sand. $r_{u}^{*}$ in the reinforced sand remains less than 0.25, and the samples are not liquefied. Being different from the VL samples, increasing FC from 0.25% to 0.5% does not yielded a significant drop in $r_{u}^{*}$, which is because PDC is initiated by the tensile stress mobilised in the fibres, causing a rise in excess pore water under large shear strains (Figure 8b). Under triaxial extension, added fibres increase the dilating tendency of the sand skeleton and thus negative EPP largely generates. $r_{u}$ of the reinforced samples with FC = 0.25% and 0.5% drops to −0.56 and −0.78 from the 0.48 (for the unreinforced sand).

The MD sand has a great tendency to dilate. Under undrained triaxial compression, large negative EPP develops in the unreinforced sand, yielding a $r_{u}$ of −1.94, where the liquefaction is absent. The tensioned fibres significantly suppress the sand skeleton’s tendency of dilation, and a PDC occurs under large $\varepsilon_{q}$, leading to a considerable rise in EPP (Figure 10b). A rise in $r_{u}^{*}$ therefore occurs with the increase in FC. Under undrained triaxial extension, $r_{u}$ in the unreinforced sand reaches −3.32. When fibres are present, the dilating tendency of the sample increases, and $r_{u}$ in the reinforced samples decreases. The liquefaction never occurs.

### 4.3. Discussion

Figure 21 shows that adding fibres to sand improves the liquefaction resistance of sand under both undrained triaxial compression and extension. The anisotropic liquefaction responses in the reinforced samples are observed under undrained triaxial compression and extension. The anisotropy is mainly sourced from the anisotropic distribution of fibre orientations, which causes the fibres to play varied roles in suppressing the liquefaction potential of the sand under different loading paths.

Under undrained triaxial compression, tensioned fibres make the reinforced sand show a greater tendency to contract than when under extension. The liquefaction resistance of sand is more likely to be increased by the confining effect. The tensile stress mobilised in the fibres alters the effective stress acting on the sand skeleton, forcing the sand skeleton to move far away from a liquefied state with the fibres being continuously tensioned. Under undrained triaxial extension, tensile stress mobilised in the fibres is quite limited. The alteration of the liquefaction response is predominantly caused by the introduced tendency of dilation by fibres (i.e., densifying effect). The greater tendency of dilation in the reinforced samples significantly reduces the build-up of positive EPP, which also forces the effective stress state of the sample to head toward a path away from the liquefied state.

## 5. Conclusions

Drained and undrained triaxial compression and extension tests were performed on unreinforced and fibre-reinforced samples in different density states (i.e., VL, L and MD). The effects of fibre reinforcement on the liquefaction resistance of sand, especially the anisotropy involved in the liquefaction resistance of the reinforced sand, were discussed. Several conclusions were drawn.

Adding fibres to sand significantly increases its strength under drained triaxial compression. Under a confining stress of 100 kPa, the drained strength of the reinforced sample with FC = 0.5% is increased by 232.2% more than the unreinforced sample at $\varepsilon_{q}$ = 40%. The strength improvement in fibre reinforcement is insignificant under drained triaxial extension. Adding fibres makes sand more contractive under drained triaxial compression while the sand shows more dilation under drained triaxial extension when fibres are added.

Fibre reinforcement improves the liquefaction resistance of sand. The presence of fibres prevents liquefaction from developing even when the sand samples are in very loose states. Under undrained triaxial compression, the fibre-reinforced samples showed strain-hardening responses irrespective of density state, while under undrained extension, the addition of fibres significantly suppresses the extent of strain-softening.

Two main effects, i.e., the densifying effect and confining effect, were introduced by the presence of fibres, which may benefit the boost in liquefaction resistance of sand. The densifying effect increases the dilating tendency of the sand, suppressing the build-up of the positive EPP. The confining effect alters the ESP of the sand skeleton, forcing the stress state of the sand skeleton to move far away from a liquefied state even though the conventional EPP ratio ($r_{u}$) approaches unity. An alternative EPP ratio, which incorporates the stress contributions of fibres, denoted as $r_{u}^{*}$, made for a better assessment of the liquefaction resistance of the fibre-reinforced sand.

Under triaxial compression, the strength envelope of the reinforced sample has a greater slope and intercept than the unreinforced sand, indicating that adding fibres improves the strength of the sand as well as the ‘cohesion’ to overcome the fragilities of the sand. The constitutive modelling framework based on the rule of mixture demonstrates the change of strength envelope of the reinforced sand is caused by the stress contributions of the fibres. The alteration of adding fibres did not significantly affect the strength envelope of the sand once triaxial extension loading had been imposed.

ESP reversal was experienced by the fibre-reinforced samples even though they were in VL and L states. Under triaxial compression, the stress ratio initiated the ESP reversal of reinforced sand drops once the samples were in a denser state. The rule of the mixture-based constitutive model demonstrates that the ESP reversal of the reinforced sand is affected by the stress contribution of fibres. The ESP reversal occurs when the increment in EPP is ‘felt’ by the sand skeleton, i.e., ${\delta \mu }_{f}p_{f}+\delta u$ attains zero, at which $\delta r_{u}^{*}$ = 0. In a denser sample, a greater tendency to dilate may suppress the generation of the positive EPP and lesser mean stress contribution of fibres may be required to balance out the EPP, and inducing ESP reversal in a denser sample occurs at a lower stress ratio. The ESP reversal experienced by the samples under undrained triaxial extension is mainly caused by the phase transformation, and adding fibres does not significantly affect the stress ratio initiating the ESP reversal.

After calculating $r_{u}^{*}$, the final liquefaction states of both unreinforced and fibre-reinforced samples were illustrated. The anisotropy in the liquefaction resistance was observed in the reinforced sand under undrained triaxial compression and extension. The anisotropy is induced by the varied roles played by the fibres in suppressing the liquefaction under different loading paths, which result from a nonuniform distribution of the fibre orientation. Under undrained triaxial compression, the liquefaction of sand is predominantly suppressed by the confining effect while under undrained triaxial extension, the liquefaction resistance of sand mainly benefits from the densifying effect.

This study did not particularly focus on the practical application of fibre-reinforcing technology. The possible application of the technology in mitigating the liquefaction potential of sand foundations should be further investigated by performing physical model tests and field tests in the future.

## Figures and Tables

**Table 1:** The index properties of Sydney sand.

Mean Grain Size, *D*_50_ (mm)	Coefficient of Uniformity, *C*_u_	Coefficient of Curvature, *C*_c_	Maximum Void Ratio, *e*_max_	Minimum Void Ratio, *e*_min_	Specific Gravity, *G_s_*
0.28	1.94	0.86	0.97	0.6	2.65

**Table 2:** The tests performed.

Test	FC (%)	*p_c_* (kPa)	Void Ratio after Consolidation	Density State
DMC-00-50-VL	0	50	1.0446	Very loose state
DMC-25-50-VL	0.25		1.0151	
DMC-50-50-VL	0.5		1.0094	
DMC-00-100-VL	0	100	1.0339	
DMC-25-100-VL	0.25		1.0124	
DMC-50-100-VL	0.5		0.9874	
DMC-00-200-VL	0	200	1.0414	
DMC-25-200-VL	0.25		1.0007	
DMC-50-200-VL	0.5		0.9966	
DME-00-100-VL	0	100	1.0408	
DME-25-100-VL	0.25		1.0212	
DME-50-100-VL	0.5		1.0000	
UMC-00-50-VL	0	50	1.0480	
UMC-25-50-VL	0.25		1.0337	
UMC-50-50-VL	0.5		0.9983	
UMC-00-100-VL	0	100	1.0446	
UMC-25-100-VL	0.25		1.0028	
UMC-50-100-VL	0.5		0.9876	
UMC-00-200-VL	0	200	1.0371	
UMC-25-200-VL	0.25		1.0004	
UMC-50-200-VL	0.5		0.9870	
UME-00-100-VL	0	100	1.0302	
UME-25-100-VL	0.25		1.0025	
UME-50-100-VL	0.5		1.0023	
UMC-00-50-L	0	50	0.9214	Loose state
UMC-25-50-L	0.25		0.9130	
UMC-50-50-L	0.5		0.8953	
UMC-00-100-L	0	100	0.9176	
UMC-25-100-L	0.25		0.8977	
UMC-50-100-L	0.5		0.8833	
UMC-00-200-L	0	200	0.8966	
UMC-25-200-L	0.25		0.8857	
UMC-50-200-L	0.5		0.8686	
UME-00-100-L	0	100	0.9204	
UME-25-100-L	0.25		0.8813	
UME-50-100-L	0.5		0.8621	
UMC-00-100-MD	0	100	0.7790	Medium dense state
UMC-25-100-MD	0.25		0.7653	
UMC-50-100-MD	0.5		0.7452	
UME-00-100-MD	0	100	0.7832	
UME-25-100-MD	0.25		0.7696	
UME-50-100-MD	0.5		0.7692	

Note: U = undrained loading; D = drained loading; MC = monotonic compression; ME = monotonic extension; VL = very loose state; L = loose state; and MD = medium dense state.

## Data Availability

All experimental data that support the findings of this study are available from the corresponding author upon reasonable request.
